# Design Strategies for Aptamer-Based Biosensors

**DOI:** 10.3390/s100504541

**Published:** 2010-05-04

**Authors:** Kun Han, Zhiqiang Liang, Nandi Zhou

**Affiliations:** 1 Department of Biochemistry and National Key Laboratory of Pharmaceutical Biotechnology, Nanjing University, Nanjing 210093, China; 2 Suzhou Institute of Biomedical Engineering Technology, Chinese Academy of Science, Suzhou 215163, China; E-Mail: hank@sibet.ac.cn; 3 Laboratory of Biosensing Technology, School of Life Sciences, Shanghai University, Shanghai 200444, China; E-Mail: lzq9130@yahoo.com.cn; 4 School of Biotechnology and the Key Laboratory of Industrial Biotechnology, Ministry of Education, Jiangnan University, Wuxi 214122, China

**Keywords:** aptamer, biosensor, design strategy

## Abstract

Aptamers have been widely used as recognition elements for biosensor construction, especially in the detection of proteins or small molecule targets, and regarded as promising alternatives for antibodies in bioassay areas. In this review, we present an overview of reported design strategies for the fabrication of biosensors and classify them into four basic modes: target-induced structure switching mode, sandwich or sandwich-like mode, target-induced dissociation/displacement mode and competitive replacement mode. In view of the unprecedented advantages brought about by aptamers and smart design strategies, aptamer-based biosensors are expected to be one of the most promising devices in bioassay related applications.

## 1. Introduction

Basically, a biosensor is a device that comprises two or more of the following parts: recognition component, linker, localization component, signal receptor, signal amplification component and signal transducer, *etc.* Among them, at least two parts are unavoidable: one is the recognition part, which could specifically recognize and identify target, and the other is the signal transduction part, which could transform the signal of analyte into some detectable signal. Recognition components, typically enzymes, antibodies or nucleic acids, directly decide the selectivity, sensitivity, stability, as well as application prospects of biosensors. Among them, antibodies were regarded as almost the only choice in protein detection. Recently, a new class of recognition components, aptamer, has attracted tremendous attention and been supposed to be promising alternatives of conventional recognition elements.

Aptamers are oligonucleotides (single-stranded DNA or RNA) that can bind their targets with high affinity and specificity. Ever since the concept of aptamer was raised by Ellington [1] and Louis [2] and the technology of systematic evolution of ligand by exponential enrichment (SELEX) was developed for isolation of aptamers in 1991 [3], more and more aptamers have been reported, which could specifically bind to various target ligands cover from small ions, single molecules to proteins, even cells. Recently, aptamers have received tremendous attention as recognition components in the biosensors design and analytical and diagnostic applications. Compared with conventional recognition components—monoclonal antibodies, aptamers possess analogical or even better attributes [4–6]:

- Aptamers show high affinity and exquisite specificity for cognate ligands as well as for some ligands which could not be recognized by antibodies, such as ions or small molecules, indicating that employing aptamers as the recognition components may markedly broaden the applications of the corresponding biosensors.
- Once selected, aptamers could be massively synthesized via chemical progress, which is more cost-effective than the production of antibodies. Consequently, the cost for fabrication of aptamer-based biosensors could be cut down.
- Aptamers can more easily be modified chemically than antibodies, especially for the modification of signal moieties, such as electrochemical probes, fluorophores and quenchers, which greatly facilitates the fabrication of biosensors.
- Aptamers are more robust at elevated temperatures, and thermal denaturation of aptamers is reversible. While as proteins, antibodies are more thermally sensitive, and denaturation of antibodies is usually irreversible. Thus use of aptamers offers a wide range of assay conditions.
- The binding of aptamers with their targets usually relies on specific conformations, such as G-quaduplex, hairpin. The conformational variations before and after the formation of aptamer-ligand complexs offer a great possibility and feasibility for the construction of aptamer-based biosensors.
- Owing to their oligonucleotide nature, they could interact with other DNA or RNA molecules, such as DNAzymes, thus fabricate versatile oligonucleotides machines for either biosensing or other clinical applications.
- Nucleic acid aptamers can hybridize with their complementary sequences, which can be used to create the antidotes.

Due to the above advantages, aptamers are regarded as promising alternatives to antibodies in bioassay related fields and have been applied in bio-molecule detection, cell collection and detection [7,8], imaging [9], or other clinical treatments [4].

Till now, several review works of aptamer-based biosensing have been published. Guo and Dong [10] presented approaches that involve nanoparticles (NPs) in electrochemical aptamer sensors together with other type of electrochemical sensors, such as genosensors, immunosensors. Willner *et al.* [11] discussed the design of aptamer-DNAzyme conjugates and their applications in amplified biosensing and logic gate operation. Lee *et al.* [12] reported the construction of nanoscale electrical biosensors using aptamers as molecular recognition elements and highlighted the advantages of aptamers over antibodies. Song *et al.* [5] exhibited an overview on the development of aptamer-based biosensors and bioassay methods. Strehlitz *et al.* [13] summarized the application of aptamers in protein detection. In Mok and Li’s review work [14], they introduced in detail the properties and the selection methods of aptamers, progress in engineering biosensors, and the prospect of chemical and biological applications. More recently, Sefah and co-workers [15] discussed the use of aptamers in biosensor development by sorting them into three general paradigms: structure-switching, enzyme-based, and aptazyme-based biosensors. While in this review, we try to give a systematic overview that concerns on the design strategies for the fabrication and application of aptamer-based biosensors. Although the detection approaches or instruments may be different for various aptamer-based biosensors, the essences of the design strategies are somewhat similar. The main design strategies will be summarized, classified and compared with detailed examples.

## 2. Strategies for the Construction of Aptamer-Based Biosensors

As is well known, aptamers interact with their targets and form special three dimensional conformations. According to the relative spatial positions of targets and aptamers in the target-aptamer complexes, the targets could be divided into two groups: embedded group and outside-binding group. The ligands belong to embedded group are usually buried within a small binding pocket formed by special oligonucleotides sequence of aptamers. Small molecules, such as ATP [16], cocaine [17], K^+^ [18] and theophylline [19], are typical targets of this group. Because of the embedment of the ligands, the design strategies mainly concern on the manipulation of the aptamers. Biomacromolecules, typically protein targets, which possess complicated spatial structures, belong to the outside-binding group. Some proteins, such as thrombin [20], platelet-derived growth factor-BB (PDGF-BB) [21], have more than one aptamer-binding site, which could offer diverse designs of sensing strategies.

Till now, plenty of research works concerned with the fabrication of aptamer-based biosensors have been reported. These biosensors were well-constructed by a variety of methodologies, including electrochemical biosensors, optical biosensors, mass-sensitive biosensors, *etc.* However, the design strategies of most of these biosensors have some versatile or analogical elements. Thus, we try to divide these strategies into four modes: (a) target-induced structure switching mode; (b) sandwich or sandwich like mode; (c) target-induced dissociation or displacement mode; (d) competitive replacement mode.

### 2.1. Target-Induced Structure Switching (TISS) Mode

In this mode, the targets directly bind to their aptamers, along with the corresponding conformational switch of aptamers to specific patterns instead of random structures, and subsequently lead to the changes of detectable characters, including:

- The position, quantity or status of the signal moieties, which covalently bind to the end of aptamers or adsorbed on the aptamers via electrostatic force, stacking, hydrogen bond, *etc.*;
- The size or weight of aptamers, along with the formation of aptamer-target complexes;
- Other properties of aptamers, such as the ability to stabilize gold nanoparticles (AuNPs), *etc.*

Each of the changes above implies a change of signals and opportunity for the design of aptamer-based biosensors. Such mode of design fits to both embedded group and outside-binding group. Several different designs of TISS mode have been reported, such as the aptamer beacon [22,23], target-responsive electrochemical aptamer switch (TREAS) [24].

Upon the binding of the targets to the aptamers immobilized on the surface of electrodes, aptamers switch into rigid tertiary structures, such as G-quaduplex, from random flexible conformations. Such conformational switches would change the relative positions of signal moieties which are covalently linked to the end of aptamers towards the electrodes, leading to the changes of electrochemical signals. Based on such TISS, several electrochemical aptamer-based biosensors have been designed.

**Figure 1.**
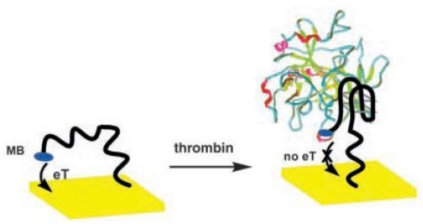
Design of a signal-off electrochemical aptamer biosensor for thrombin detection (adapted from reference [20], with kind permission).

A redox probe-labeled, signal-off electrochemical biosensor was first developed for thrombin detection [20]. Before thrombin binding, the electrochemical active redox moiety, methylene blue (MB) which was labeled at the end of aptamer covalently, could transfer electron with the electrode surface due to the flexible conformation of the aptamer. However, once thrombin was captured by the aptamer and the G-quaduplex structure was formed, the MB moiety was kept away from the electrode surface, resulting in the electrochemical signal-off (Figure 1). Such design has a main disadvantage of negative signal.

**Figure 2.**
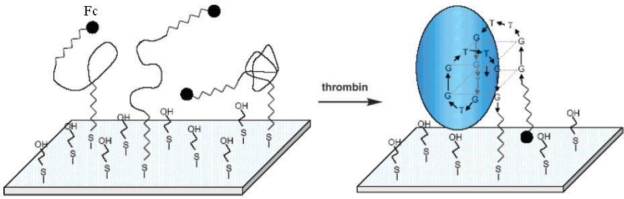
Design of a signal-on electrochemical aptamer biosensor for thrombin detection (adapted from reference [22], with kind permission).

To overcome aforementioned disadvantage, signal-on electrochemical biosensors were developed. In a typical work, thrombin-binding aptamer immobilized on polycrystalline gold electrode was covalently attached with ferrocene (Fc) at the other end [22]. The electrode surface was further blocked by 2-mercaptoethanol. No signal was obtained before the binding of thrombin, because the flexible unfolded aptamer led to the large electron-tunneling distance between Fc moiety and the electrode. In the presence of thrombin, the rigid G-quaduplex structure would drag the Fc moiety to approach the electrode surface, and the electron transfer between the Fc and electrode was realized (Figure 2). The signal-on design of this biosensor produces positive signals, which apparently broaden the detection range of biosensors. However, the flexibility of aptamers immobilized on electrode surface can bring high background signals and influence the reproducibility of biosensors.

To circumvent the problems induced from random structure of single-stranded oligonucleotides, the immobilized aptamer with Fc label was hybridized with its complementary sequence to form double-stranded DNA (dsDNA). In the absence of target, electron transfer between Fc at the end of aptamer and the electrode was completely blocked due to the large distance and the rigid structure of dsDNA. Thus the background signal could be effectively inhibited. However, in the presence of target, the complementary sequence was liberated and the aptamer switched to hairpin structure and bound with the target, which greatly shortened the distance between Fc and the electrode and subsequently turned on the electron transfer path. This biosensing strategy was named TREAS [24]. In this case, the design of the complementary sequence of the aptamer is important. The dissociation constant of aptamer and its complementary sequence, together with the dissociation constant of aptamer and its target would markedly influence the performance of biosensors.

Similar research has also been published. The fabricated biosensors have been used for the detection of thrombin [23,25], theophylline [19], PDGF [26,27], cocaine [28,29], not only for standard samples, but also in clinical samples [24,26].

**Figure 3.**
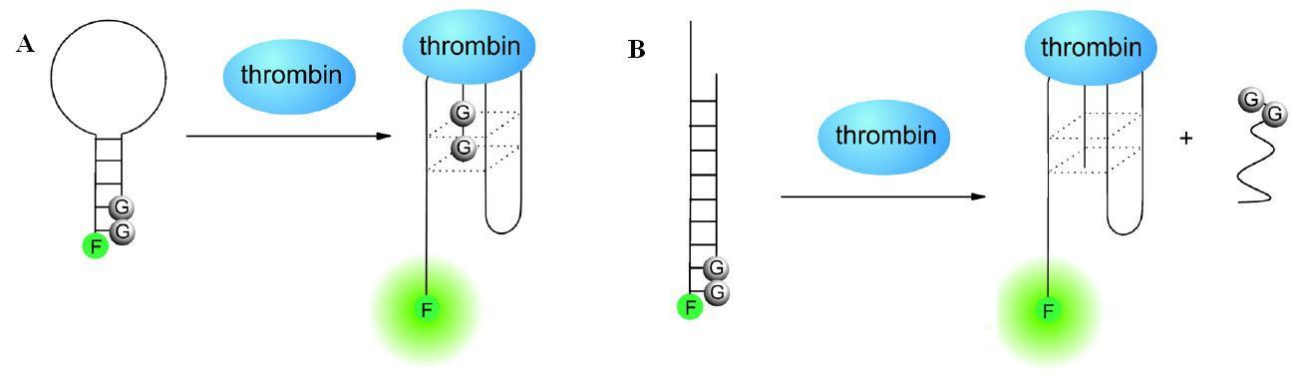
Schemes for the target-induced structure switching aptamer beacons. (A) Hairpin design and (B) duplex design (adapted from reference [30], with kind permission).

Besides electrochemical biosensors, TISS strategy could utilize to design other classes of biosensors. Aptamer-based molecule beacon fluorescent detection is another frequently utilized way for biosensor design. In aptamer beacons, fluorophores which can provide report signals and quenchers which can quench fluorescent signals are covalently linked to the ends of aptamers or the complementary sequences of aptamers. The relative position of fluorophores and quenchers, which decides the intensity of fluorescent signal, is related with the presence of the target, and subsequently the conformation of aptamer. For instance, guanine bases were used as quencher to develop two different cost-effective and label free aptamer-based sensors, named hairpin design and duplex design [30]. In the absence of target thrombin, the aptamer sequence formed a hairpin structure by itself in the hairpin design (Figure 3A) or double-stranded status with a complementary sequence in the duplex design (Figure 3B). The 5′-end fluorescent signal moiety was quenched by the guanine bases at the 3′-end of the aptamer or the 3′-end of complementary sequence, hence, quenching structures were formed and no fluorescent signal could be detected. However, in the presence of thrombin, the formation of target-aptamer complex would destruct the quenching structure and separate the fluorophore from the guanine bases, thus restore fluorescence signal. In another research, pyrene monomers were labeled with each end of the aptamer of PDGF. While the target existed, the fluorescence emission of the labeled aptamer switched from 400 nm to 485 nm, due to the conformational rearrangement of aptamer induced by target binding. The established method has been proven to be effective in direct detection and quantification of PDGF in complex biological samples [31].

**Figure 4.**
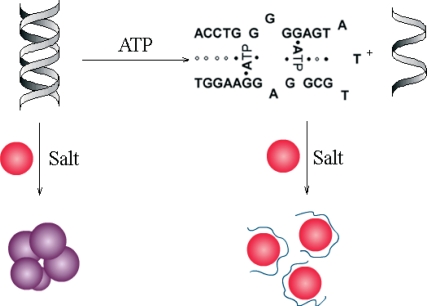
Scheme for the target-induced structure switching and salt-induced AuNPs aggregation (adapted from reference [34], with kind permission).

Colorimetric assays are another popular optical detection technique. In recent years, AuNPs have been widely used in optical biosensor design, due to their characteristic spectroscopic changes during target-induced aggregation or dispersion. Researchers showed that adsorption of single-stranded DNA (ssDNA) on AuNPs can effectively stabilize the colloid against salt-induced aggregation while dsDNA has no such function [32,33]. Then colorimetric aptamer-based biosensors were developed [34]. While the target ATP was absent, the aptamer hybridized with its complementary sequence to form a rigid duplex, which could not prevent AuNPs from aggregating as salt added into the system. However, as ATP was present, the duplex structure would switch to ATP-aptamer complex together with the release of its complementary sequence, which could stabilize AuNPs and show high resistance to salt-induced aggregation (Figure 4). The changes of solution color caused by AuNPs aggregation could easily observed by naked eyes. And the corresponding changes in UV-visible spectra could be used to quantitative assay of ATP. Similarly, another research indicated that the AuNPs modified by target-binding folded aptamer showed higher stability toward salt-induced aggregation than the AuNPs modified with unbinding aptamer. And a colorimetric biosensor was developed for the assay of ATP or K^+^, according to this target-induced structure switching of the aptamer modified on the surface of AuNPs [18].

Besides the electrochemical and optical biosensors mentioned above, mass-sensitive biosensors could also be fabricated via TISS strategy, which detect target-induced changes in size or mass of aptamers. Frequently used mass-sensitive techniques include surface plasmon resonance (SPR) [35], quartz crystal microbalance (QCM) [36] and surface acoustic wave device (SAW) [37], *etc.* In an aptamer-based AuNPs-enhanced SPR detection, AuNPs, the SPR signal enhancer, was immobilized via DNA hybridization with the aptamer sequence. Like the TREAS strategy mentioned above, the binding of ATP led to formation of tertiary structure and dehybridization between aptamer and the AuNPs tagged complementary ssDNA, which finally resulted in the decrease of the SPR signal [35].

### 2.2. Sandwich or Sandwich-Like Mode

As named, the sandwich mode devices consist of at least three parts, “a piece of meat” inserted within “two pieces of bread”. Some protein targets, such as PDGF-BB and thrombin, have dual binding sites, which endows them the ability to bind two recognition molecules and form sandwich-like complexes. Since these proteins could specifically bind either aptamers or antibodies, the sandwich structures have three basic formats: aptamer-protein-aptamer, aptamer-protein-antibody and antibody-protein-antibody. The first two formats have been widely employed in design of aptamer-based biosensors.

**Figure 5.**
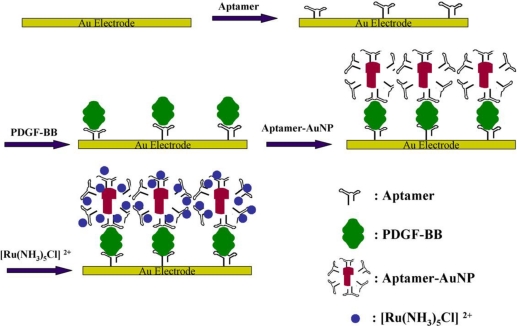
Scheme for the formation of sandwich structure and Au-NPs mediated amplification for PDGF detection (adapted from reference [21], with kind permission).

An electrochemical biosensor for PDGF detection via sandwich structure and AuNPs mediated amplification has been constructed [21]. As shown in Figure 5, the aptamer was immobilized on the electrode surface through self-assembly. In the presence of the target PDGF-BB, it would be captured onto the interface through the formation of PDGF-aptamer complex. Then the aptamer modified AuNPs, which are negative charged, recognized the target specifically and bound to the electrode surface to form a sandwich structure. Finally, the positive charged [Ru(NH_3_)_5_Cl]^2+^, used as probe, was adsorbed to the sandwich structure via electrostatic interaction. The obtained electrochemical signals were directly concerned with the concentration of PDGF.

Similarly, sandwich mode has been reported for the high specific detection of thrombin through construction of aptamer-protein-aptamer structure in the electrochemiluminescence (ECL) sensor design [38], the magnetic beads coupled assay [39] and the impedimetric assay [40]. Meanwhile, antibodies were also employed for the recognition of target, together with aptamer for the signal carrier, to detect thrombin through the formation of aptamer-protein-antibody structure [41,42].

**Figure 6.**
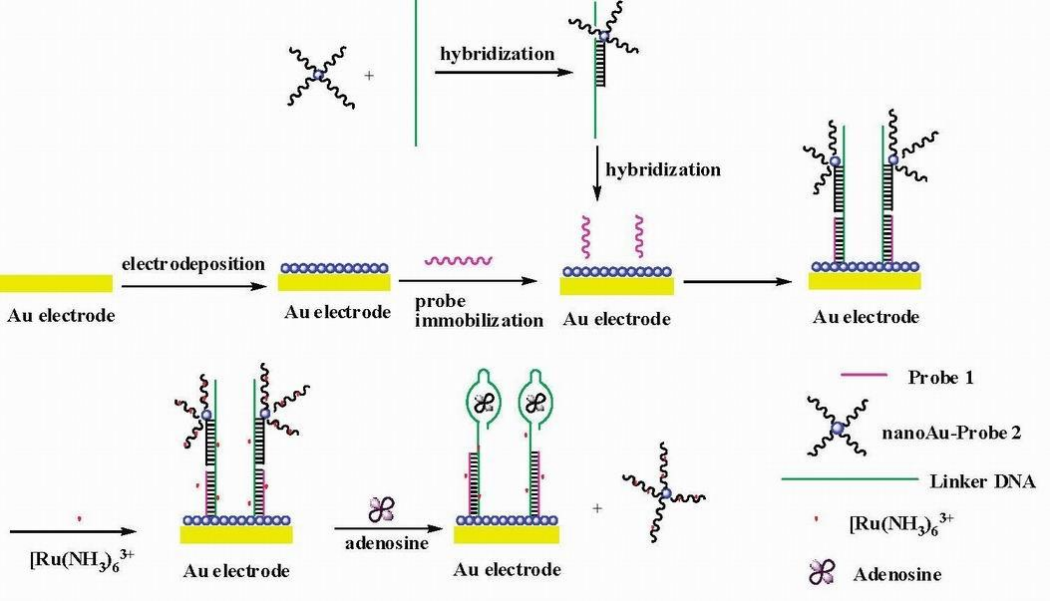
Scheme for sandwich-like structure for the detection of adenosine (adapted from reference [43], with kind permission).

Since the sandwich design requires dual binding sites of targets, it can hardly be applied to the assay of embedded groups of targets. However, another type of sandwich-like mode can be adapted to their detection, which involves the manipulation and design of the aptamers and their complementary sequences. For instance, in an adenosine biosensor design [43], a thiolated capture probe was immobilized onto the AuNPs modified gold electrode. Then a linker DNA containing antiadenosine aptamer sequence and reporter DNA loaded on AuNPs was bound to the electrode through hybridization. [Ru(NH_3_)_6_]^3+^ was adsorbed to DNA molecules via electrostatic interaction and used to produce electrochemical signal. In the presence of adenosine, the aptamer part of the linker DNA bound with adenosine to form target-aptamer complex structure, resulting in the release of reporter probes modified AuNPs together with [Ru(NH_3_)_6_]^3+^, which subsequently led to the decrease in electrochemical signal (Figure 6). Based on similar mechanism, an ATP biosensor fabricated via fluorescence resonance energy transfer (FRET) between a quantum dot as donor and an organic fluorophore as acceptor has also been reported [44].

### 2.3. Target-Induced Dissociation/Displacement (TID) Mode

Both the TISS mode and the sandwich mode belong to structure-dependent assay. The fabrication of those aptamer-based biosensors is highly relying on the special conformations of the aptamers (such as G-quaduplex or hairpin conformation), or the specific target-aptamer complex structures. However, in the design of some biosensors, especially for the electrochemical aptamer-based biosensors, a TID mode, which belongs to a structure-independent strategy, has been raised [16].

**Figure 7.**
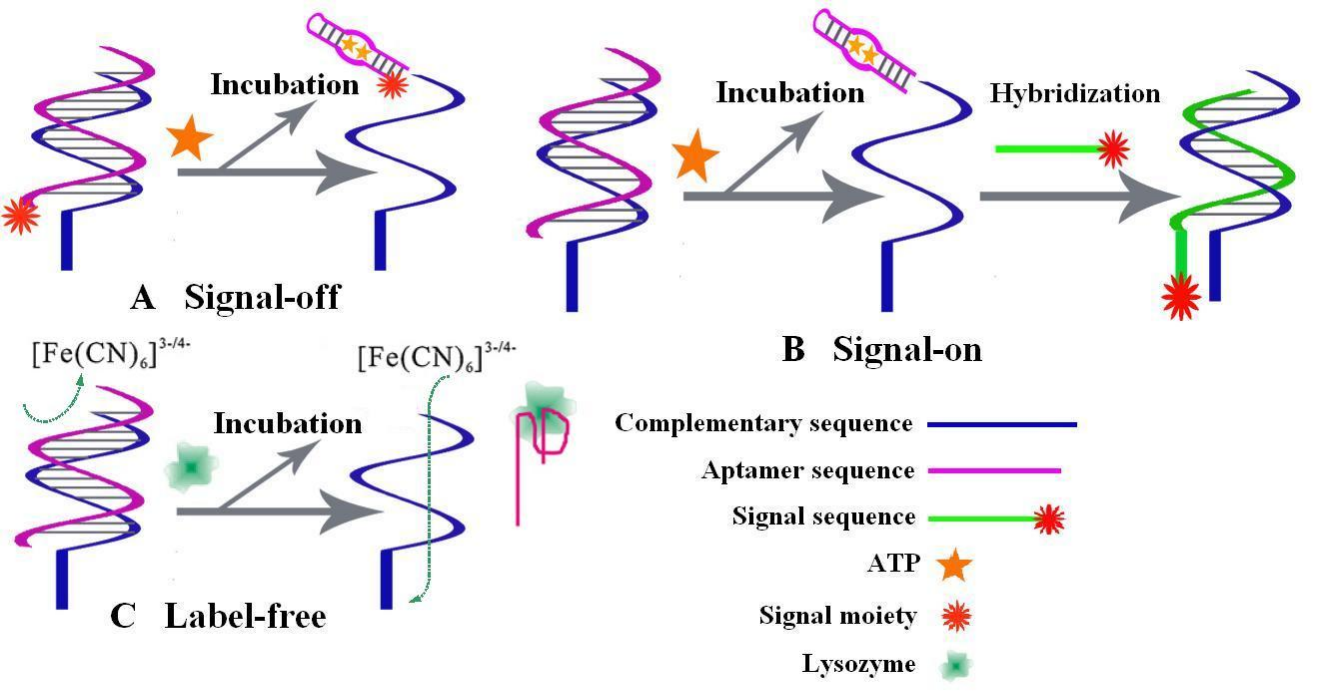
Schemes for the target-induced dissociation/displacement strategies. **A.** signal off mode [45]; **B** signal-on mode [16]; **C** label-free mode [50].

In TID mode, the complementary sequences of aptamers, instead of aptamers themselves, are employed as anchors to localize the aptamers. After incubation with targets, the formed target-aptamer complexes will be liberated into the solution, which leads to the changes of detectable signals. TID strategy can be further classified into signal-off mode [45–47], signal-on mode [16,48] and label-free mode [49,50], as shown in Figure 7.

In signal-off mode, the presence of targets can be determined from the signal suppression due to the release of redox-tagged aptamer from the electrode into solution. As an example, signal-off design for ATP and thrombin assay [45], the redox-tag attached aptamers was confined near the electrode surface through the hybridization with their thiolated complementary sequence, which was self-assembled on the gold electrode. In the absence of targets, the redox-tag gave electrochemical signals. While targets were added, the redox-tagged aptamers were released and the electrochemical signals were greatly reduced (Figure 7A). Then signal-on mode TID strategy has also be developed. In the construction of an ATP biosensor [16], the aptamer hybridized with its thiolated complementary sequence was immobilized on the gold electrode via Au-S bond. As the target ATP was added, the aptamer sequence was released from double strands to form target-aptamer complex. The the remained complementary sequence was further hybridized with another Fc-moiety modified ssDNA, resulting in the emergence of electrochemical signal (Figure 7B). Further, label-free TID strategy was also proposed. In a faradic impedance spectroscopy (FIS) for lysozyme assay [50], the release of the aptamer due to the formation of target-aptamer complex would decrease the interfacial electron transfer resistance, which was utilized to quantify the concentration of lysozyme (Figure 7C).

Besides electrochemical biosensors, the TID mode has also been widely employed in other types of aptamer-based biosensors. For example, in an adenosine detection method, the target induced the dissociation of aptamer from its complementary signal sequence, which could be captured later and characterized via surface-enhanced Raman scattering method [51]. Similarly, a surface enhanced resonance Raman scattering (SERRS) biosensor for thrombin detection has also been fabricated [52]. For colorimetric assay, the presence of adenosine induced the dissociation of aptamer from dsDNA modified AuNPs and the remained ssDNA modified AuNPs would aggregate, which could be observed by the naked eye [53].

### 2.4. Competitive Replacement Mode

As aptamers share similar characteristics with antibodies, various detection methods which take advantage of antibodies can be developed into aptamer-based methods. For instance, most immunoassays for small molecules are competitive assays relying on the replacement of surface-bound antibodies by the analyte in solution. This replacement mode could be applied to the aptamer-based assays. Hence a new type of strategies, named as competitive replacement mode, has been developed. In this mode, signal modified target molecules need to be designed and synthesized in advance.

**Figure 8.**
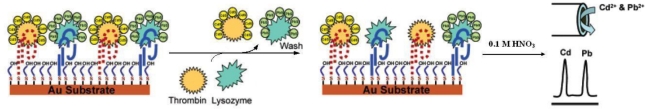
Dual-analyte biosensor designed via the competitive replacement mode and electrochemical stripping detection (adapted from reference [55], with kind permission).

As early as in 2000, a fiber-optic microarray biosensor using aptamers as receptors was developed [54], which was complementary to the conventional ELISA technique. In this research, the aptamer bead on the fiber surface was incubated with the fluorescence-labeled thrombin (F-thrombin). As the non-labeled target thrombin was added, it would competitively bind to the aptamer bead and replace the F-thrombin, which led to the decrease of fluorescent signal. Hence, non-labeled thrombin could be detected. In recent years, nanoparticles, especially quantum-dots (QDs) have been employed to label proteins. As shown in Figure 8, an ultra-sensitive electrochemical biosensor has been developed for the detection of thrombin and lysozyme simultaneously [55]. The thiolated aptamers of thrombin and lysozyme were co-immobilized onto the gold substrate, followed with the binding of CdS-labeled thrombin and PdS-labeled lysozyme through the formation of labeled protein-aptamer complexes respectively. As the sample containing detection targets was added, the label-proteins could be displaced by targets, which could be characterized through electrochemical stripping detection.

Recently, the competitive replacement mode has been applied to the detection of the small molecule neomycin B in milk via the FIS [56] and SPR [57] approaches. Neomycin B was immobilized on a self-assembled monolayer (SAM) of mercaptopropionic acid on a substrate surface through carbodiimide chemistry. Then, the aptamer was attached to the neomycin B via binding affinity. The presence of neomycin B in sample would competitively bind to the aptamer modified on the surface, leading to changes in surface modification, which could then be detected via FIS or SPR.

Carbon nanotubes (CNTs) have widely attracted substantial research interests due to their special structural features and unique electronic properties, and have also been applied in aptamer-based biosensing. For example, single-walled carbon nanotubes (SWCNTs), with the ability to quench covalently tethered and/or π-stacked pyrenes, porphyrins and chromophores [58], have been used to establish a sensing platform in which the targets could competitively bind to the dye-labeled aptamer. As a result, SWCNTs were substituted and the quenching effect was eliminated. Thus, the recovered fluorescence signal of the dye molecules could be used to detect targets [59].

### 2.5. Aptamer-Based DNA/RNA Machine in Biosensor Design

#### 2.5.1. Aptamer-aptamer combination

An aptameric enzyme subunit (AES) is a DNA aptamer composed of an enzyme-inhibiting aptamer and a target-binding aptamer. It can allosterically regulate the corresponding enzymatic activity upon binding to the target molecule, which means, measuring the corresponding enzymatic activity could indirectly determine the target molecule in a homogeneous solution. AESs were originally designed for detection of insulin [60], IgE [61] and adenosine [62] by connecting the target-binding aptamer sequences with the splinted thrombin-inhibiting aptamer. In this kind of protocol, the target molecules competitively bound to thrombin binding AESs. As a result, the AESs’ conformation changed and the allosteric regulation led to the dissociation of thrombin from the AES-thrombin complex into the solution. Using AESs, the detection of targets were realized by measuring thrombin enzymatic activity in the solution, which enabled simple and high-sensitive detection of target molecules in a homogeneous assay.

#### 2.5.2. Aptazyme

The discovery of RNAzyme and DNAzyme unraveled the innate potential of oligonucleotides in many biological applications other than as the storage media of genetic information [15]. DNAzyme/RNAzyme, as two classes of nucleic acids which have catalytic activities and special functions in biological research, have also attracted great attention for their analytical and various other applications [63]. The nature of nucleic acids endows aptamer and DNAzyme/RNAzyme native ability to cooperate with each other. For example, anti-hemin aptamer shows peroxidase activity when hemin is captured and the G-quaduplex structure is formed. Such DNAzyme could catalyze the oxidation of 2,2′-azino-bis(3-ethylbenzothiazoline- 6-sulfonic acid), ABTS^2−^, by H_2_O_2_ to the ABTS^−•^ colored product or stimulate the generation of chemiluminescence in the presence of H_2_O_2_/luminol [64]. An optical biosensor for the determination of adenosine has also been constructed via rational design. In the presence of adenosine, the anti-adenosine sequence formed a rigid structure with its ligand. The rigid structure produced a spatial hindrance and inhibited the formation of hemin-contained DNAzyme. However, the absence of adenosine would lead to the formation of DNAzyme which gave detectable signal [63]. Similar work was reported for the detection of lysozyme [64]. In another colorimetric design, by combination of anti-adenosine aptamer with DNAzyme with phosphodiester cleavage activity, the presence of adenosine could be characterized through the dispersal of aggregated AuNPs linked by the substrate strand of DNAzyme, which was inactive in the absence of adenosine [65].

## 3. Conclusions

Owing to their excellent characteristics, aptamers have been regarded as one of the most promising recognition elements in biosensor fabrication, especially in proteins or small molecules assays. An overview of the design strategies for the fabrication of aptamer-based biosensors has been presented in this review. Regardless of the kinds of targets or transducers, most reported aptamer-based biosensors can be classified into four basic modes: target-induced structure switching mode, sandwich or sandwich-like mode, target-induced dissociation/displacement mode and competitive replacement mode, which represent the dominating design strategies of such type of devices at present. Besides, several new strategies have arisen from the combination of aptamer with other functional nucleic acids, such as aptamer or the DNAzyme/RNAzyme.

Generally, these design strategies target turning the specific binding process between ligands and aptamer into different signal variations so that ligands could be effectively detected. All designed biosensors have excellent performance in the detection of standard samples. However, only a few of them have been applied in detection of real samples, such as serum or blood. Among them, TID mode shows less dependence on the conformation of the ligands or aptamers thus is more generalized compared to other strategies. There remains plenty of work to be done in this stage before aptamer-based biosensors could be practically applied, including improvements of their stability and reliability, especially in real sample detection.

On the other hand, only a small range of targets have presently been detected using aptamer-based biosensors. The extensive development of these biosensors needs to further screen and characterize the specific aptamer sequences of different targets. Nevertheless, an aptamer database has been created to contain comprehensive sequence information on aptamers and other functional nucleic acids generated by *in vitro* selection. The database is updated monthly and is publicly available at http://aptamer.icmb.utexas.edu/ [66]. As the quantity of reported aptamer sequences increases, more and more aptamer-based biosensors with excellent performances will be fabricated via smart designing strategies, which will endow them great opportunity to be applied in the fields of bioassay, biomedical diagnostic or environmental monitoring.
